# Engagement in community music classes sparks neuroplasticity and language development in children from disadvantaged backgrounds

**DOI:** 10.3389/fpsyg.2014.01403

**Published:** 2014-12-16

**Authors:** Nina Kraus, Jane Hornickel, Dana L. Strait, Jessica Slater, Elaine Thompson

**Affiliations:** ^1^Auditory Neuroscience Laboratory, Department of Communication Sciences and Disorders, Northwestern UniversityEvanston, IL, USA; ^2^Department of Otolaryngology, Neurobiology & Physiology and Northwestern University Interdepartmental Neuroscience Program, Northwestern UniversityChicago, IL, USA; ^3^Data Sense LLCChicago, IL, USA

**Keywords:** low socioeconomic status/poverty, community music training, electrophysiology, reading, speech, auditory training

## Abstract

Children from disadvantaged backgrounds often face impoverished auditory environments, such as greater exposure to ambient noise and fewer opportunities to participate in complex language interactions during development. These circumstances increase their risk for academic failure and dropout. Given the academic and neural benefits associated with musicianship, music training may be one method for providing auditory enrichment to children from disadvantaged backgrounds. We followed a group of primary-school students from gang reduction zones in Los Angeles, CA, USA for 2 years as they participated in Harmony Project. By providing free community music instruction for disadvantaged children, Harmony Project promotes the healthy development of children as learners, the development of children as ambassadors of peace and understanding, and the development of stronger communities. Children who were more engaged in the music program—as defined by better attendance and classroom participation—developed stronger brain encoding of speech after 2 years than their less-engaged peers in the program. Additionally, children who were more engaged in the program showed increases in reading scores, while those less engaged did not show improvements. The neural gains accompanying music engagement were seen in the very measures of neural speech processing that are weaker in children from disadvantaged backgrounds. Our results suggest that community music programs such as Harmony Project provide a form of auditory enrichment that counteracts some of the biological adversities of growing up in poverty, and can further support community-based interventions aimed at improving child health and wellness.

## INTRODUCTION

Over 16 million children in the US live in families with incomes below the federal poverty level, with a disproportionate number (68%) being members of minority racial and ethnic groups (Jiang et al., 2014). Parental income, occupation, and education are commonly combined to define a child’s socio-economic status [SES (Sirin, 2005)]. A child’s SES can interact with other personal factors (race, ethnicity, gender, etc.) to lead to weaker academic achievement (Bradley and Corwyn, 2002; Sirin, 2005; Hernandez, 2011; Skoe et al., 2013) and lower high school graduation rates (Ensminger and Slusarcick, 1992; Bradley and Corwyn, 2002), with lower academic achievement persisting even for low SES students who attend college (Walpole, 2003).

Auditory processing skills, known to be important for language development (Benasich et al., 2002, 2008; Boets et al., 2011; Goswami et al., 2011), may contribute to the link between SES and academic achievement. Children from low SES backgrounds have greater daily exposure to noise (Adler and Newman, 2002; Evans and Kantrowitz, 2002) and tend to be less concerned with using hearing protection in excessively noisy contexts like concerts (Vogel et al., 2007). Chronic noise exposure in children has been linked to weaker reading proficiency and cognitive skills (Maxwell and Evans, 2000; Haines et al., 2001; Clark et al., 2005) and can lead to delayed auditory neural development and greater spontaneous neural activity in animals (Chang and Merzenich, 2003; Seki and Eggermont, 2003; Zhu et al., 2014). Additionally, children from low SES backgrounds hear less complex language and fewer words overall during early language development, which contributes to weaker vocabularies when entering school (Bradley and Corwyn, 2002; Hoff, 2003; Cartmill et al., 2013). Similarly, a host of neural systems important for language, memory, and cognition are impacted by SES background (Raizada et al., 2008; Hackman et al., 2010; Noble et al., 2012), including those implicated in auditory attention (D’Angiulli et al., 2008; Stevens et al., 2009) and speech encoding (Skoe et al., 2013). Encouragingly though, community and home based interventions may reverse these effects; children from low SES families participating in Head Start and attention training with their parents showed improved language, cognition, and neural measures of auditory attention relative to children in Head Start programs alone (Neville et al., 2013).

Musical training is another avenue of enrichment that may counteract some of the auditory deprivation endemic to low SES environments. A number of studies have revealed that children undergoing music training have stronger cognitive abilities, vocabulary, rhythm perception and production (linked to reading skill), perception of vocal pitch, and perception of speech in noisy backgrounds than non-musician children (Ho et al., 2003; Schellenberg, 2004, 2006; Magne et al., 2006; Forgeard et al., 2008b; Hyde et al., 2009; Moreno et al., 2009, 2011; Strait et al., 2011; Dege and Schwarzer, 2012; Strait et al., 2012; Slater et al., 2013; Chobert et al., 2014; Seither-Preisler et al., 2014; Strait and Kraus, 2014). Additionally, musical practice can strengthen children’s auditory encoding of speech (Magne et al., 2006; Besson et al., 2007; Chobert et al., 2011; Strait et al., 2011, 2013; Tierney et al., 2013; Chobert et al., 2014; see Strait and Kraus, 2014 for a review), auditory discrimination and attention (Koelsch et al., 2003; Moreno et al., 2009; Chobert et al., 2011; Putkinen et al., 2013), and lead to structural changes in auditory cortical areas (Hyde et al., 2009; Seither-Preisler et al., 2014). The auditory benefits of music training have direct implications for language skills and academic achievement (Hetland and Winner, 2010; Corrigall and Trainor, 2011; see Tierney and Kraus, 2013 for a review); accordingly, music may serve as an effective training tool for children with learning and attention impairments (Overy, 2003; Bhide et al., 2013; Seither-Preisler et al., 2014).

Community music programs, such as El Sistema and Harmony Project, provide students from low SES backgrounds with music opportunities that enrich the students and their communities. El Sistema, founded almost 40 years ago, provides over 500,000 Venezuelan children with free musical training in their community (for a review of the program, see Majno, 2012). Children are commonly enrolled as young as 2 or 3 years old and are supported through their teen years. Graduates of the program commonly return to teach at their community music center and parents are continually educated by the organization on how to support and encourage their child’s music as they advance. Since 2009, 62 El Sistema inspired music programs have been started in the United States. Harmony Project (Los Angeles, CA, USA) similarly promotes the development of healthy children and communities by providing free music training to children from low SES backgrounds in gang-reduction zones of Los Angeles, CA, USA (http://www.harmony-project.org). Children learn basic music skills in a preparatory class, eventually receive a free instrument to participate in group classes, and have opportunities for performance and ensemble playing throughout their enrollment from early grade school to high school. Between 2010 and 2014, 93% of Harmony Project alumni enrolled in post-secondary education, versus 67.6% of students graduating from public schools within Los Angeles County [most recent data from California Department of Education: Educational Demographics Unit (2008–2009) and Harmony Project Whole Notes 2013–2014 (2014)].

Our laboratory has shown that participation in music training through Harmony Project can reinforce literacy skills, enhance the perception of speech in background noise, and strengthen the neural encoding of speech sounds in children from low SES backgrounds (Kraus et al., 2014a,b; Slater et al., 2014a,b; Kraus and Strait, in press). Here we explore how the extent of student *engagement* in instrumental classes mediates music training’s effects on neural speech processing. Although we do not employ an active control group, our aim is to show *within a group undergoing musical training* how engagement may influence the benefits seen for students. Music engagement was defined by a student’s percent attendance in class and teacher ratings of classroom participation. We investigated whether greater engagement in music instruction can positively impact the neural encoding of speech. We focused on measures of subcortical brain activity reflecting speech harmonics, the consistency of the response, and spontaneous neural activity, all of which are weaker in teens from low SES backgrounds relative to higher-SES peers (Skoe et al., 2013).

## MATERIALS AND METHODS

### PARTICIPANTS

Twenty-six children participated in the study (13 males, ages 6 years 11 months – 9 years 3 months; *M* = 8 years 5 months). All children were public elementary school students living in the gang reduction zones of Los Angeles, CA, USA. Participants attended schools where ≥90% of students qualify for free or reduced lunch. Families qualify for free or reduced lunch when their income is below 185% of the federal poverty level (U.S. Department of Education, 2014). The average education of the participants’ mothers was 10.7 years (SD = 4.2) and the median and modal maternal educational attainment level was completion of high school/GED. Maternal education is one of the strongest predictors of SES (Hoff et al., 2012), and 76% of children whose parents have a high school degree or less live in low-income families (Jiang et al., 2014). Average maternal education was equivalent across the four Harmony Project sites in which students were enrolled (Kruskal Wallis Test: χ^2^ = 6.252, *p* = 0.100).

Participants were excluded if they had a history of neurological disorders, IQ less than 80 [Wechsler Abbreviated Scale of Intelligence (Woerner and Overstreet, 1999)], previous musical training, or failed a hearing screening (air-conduction thresholds above 20 dB HL for octaves 125–8000 Hz). All procedures were approved by the Northwestern University Institutional Review Board and informed parental consent was obtained for all participants.

### MUSIC INSTRUCTION

Students were followed as they participated in Harmony Project for 2 years. The project curriculum started students in music appreciation class, where they learned pitch-matching and rhythm skills, musical styles and notation, and basic vocal performance and recorder playing. Participants attended these classes twice weekly for 3–10 months (*M* = 5).

As musical instruments became available and students were judged to be ready, they progressed to instrumental instruction. Students were given their own instruments and participated in a mix of group-based instrumental classes for approximately 4 h per week. Students in this study participated in one of four Harmony Project sites. The description of weekly classes at each site and the number of students at each site is presented in **Table 1**. Students played a number of different instruments (viola, cello, bass, French horn, clarinet, or trumpet) and the number of students playing each type of instrument is also reported in **Table 1**. Harmony Project has a standard curriculum for each instrument type (woodwinds, brass, and strings), including rigorous mastery benchmarks required for advancement to the next level of the curriculum. For example, Level 1 benchmarks require students to: demonstrate basic knowledge of music concepts such as rhythm and intonation, exhibit instrument specific skills and knowledge such as proper posture and correct clef identification, and present evidence of commitment through practice at home. As students progress, expectations shift to more instrument specific skills (e.g., proper bowing or breathing techniques) and knowledge and use of dynamics, key signatures and deviations, and articulations (e.g., staccato). Students are required to learn to read music and play from memory throughout the program. All students in the study transitioned to instrument use during their first year, so at the end of their second year in Harmony Project, students had an average of 165 h of school-based instrumental training (SD = 40) and over 210 h of music training overall.

**Table 1:** Student instrument experience by site and by instrument.

Harmony Project site	Typical class participation	Number of students by instrument type
Alexandria elementary school	-1 h instrumental class twice per week-2 h string ensemble rehearsal weekly	4 (bass)
Beyond the bell	-2 h ensemble rehearsals twice per week (includes pull-out sectional rehearsals)	12 (2 clarinet, 3 flute, 7 trumpet)
EXPO enter (YOLA)	-1 h instrumental class each week-3 h ensemble rehearsal weekly	5 (2 cello, 1 French horn, 1 viola, 1 trumpet)
Hollywood	-1 h instrumental class twice per week-3 h ensemble rehearsal (concert band) weekly	5 (trumpet)

*Participants were enrolled in music classes in one of four sites and played one of seven different instruments. Details about the standard class schedule of each site is included*.

#### Teacher ratings of music engagement

At the end of each instrumental class, teachers reported the percent attendance for each student (hours attended/total hours possible) and rated the students on their level of participation in class (1-not at all, 2-little, 3-moderate, 4-fairly good, 5-complete). We averaged percent attendance and teacher ratings of class participation across all the instrumental classes taken by each child over the 2 years. Both attendance and teacher perception of student effort predict ‘behavioral engagement’ in school, reflecting a student’s involvement in the participatory aspects of school (Glanville and Wildhagen, 2007). Because neither percent attendance nor participation were significantly correlated with the number of instrumental classes taken (*p*-values > 0.310), we are confident that higher levels of class participation or attendance reflect student motivation and are not simply artifacts of being enrolled in more classes. Additionally, percent attendance and participation ratings did not differ across Harmony Project sites (Kruskal Wallis Test: χ^2^ = 5.366, *p* = 0.147; χ^2^ = 4.781, *p* = 0.189, respectively), suggesting no rating bias among the sites.

### READING ASSESSMENT

Before enrolling in Harmony Project and after 2 years of Harmony participation, students were administered the Test of Oral Word Reading Efficiency (TOWRE; Torgesen et al., 1999). The TOWRE, a measure of reading fluency, comprises word and non-word reading subtests. Children are required to read a list aloud as quickly as possible and the number of words (or non-words) read correctly in 45 s is tallied and combined to form the Total score.

### NEUROPHYSIOLOGICAL MEASURES

Evoked potentials were collected from the auditory brainstem in response to a 40-ms synthesized speech syllable [da], using an Intelligent Hearing Systems SmartEP system equipped with a cABR module (Miami, FL, USA). Stimuli were presented to the right ear at 80 dB SPL at a rate of 10.9 Hz through electromagnetically-shielded earphones (ER-3A, Etymotic Research, Elk Grove Village, IL, USA). Responses to opposing polarities were subtracted and filtered online from 0.1 to 1.5 kHz in the test session before music training began and from 0.05 to 3 kHz in the test session after 2 years of music training. Due to the differences in filter settings, which were expanded as the longitudinal project evolved, within-subject comparisons over the two test dates are not possible.

**FIGURE 1:**
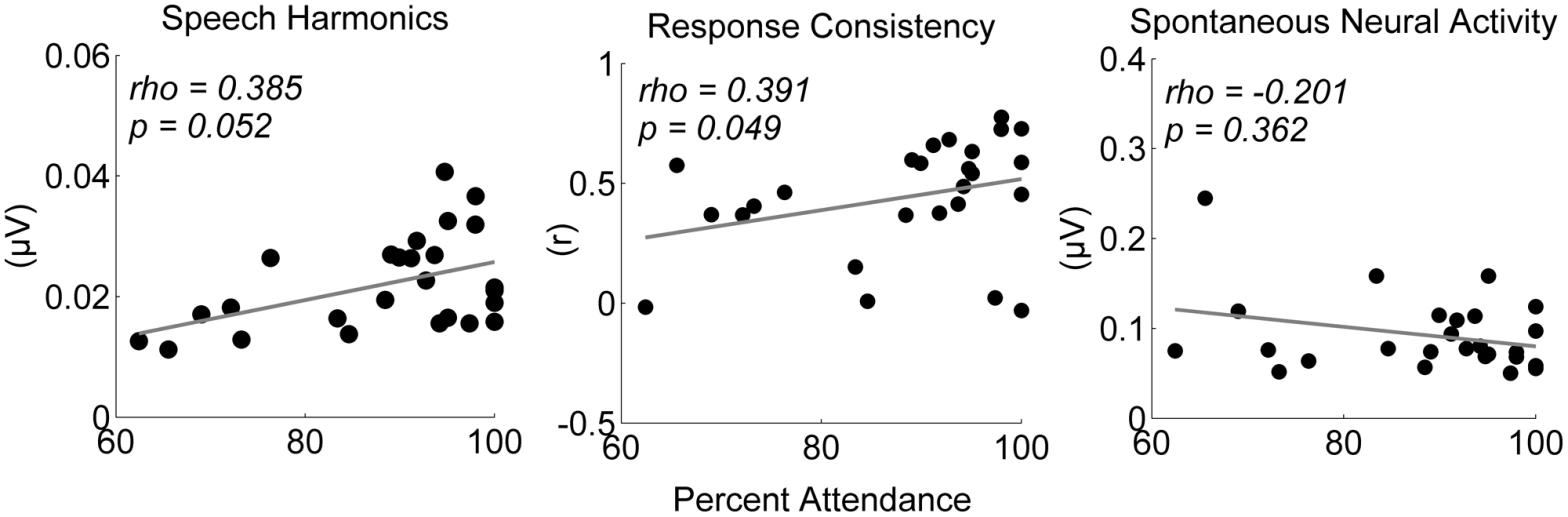
**Children who regularly attended instrumental classes had stronger neural encoding of speech after 2 years, particularly for measures of speech harmonics and response consistency.** Neural measures before music training began did not predict attendance or level of class participation, suggesting greater engagement in music classes may lead to stronger neural encoding of speech and not vice versa. Speech Harmonics is measured as the average amplitude of harmonic encoding in the frequency following response (μV), Response Consistency is measured as the Pearson’s correlation coefficient between response replications (r), and Spontaneous Neural Activity is measured as the root-mean-square magnitude of the pre-stimulus (μV).

**FIGURE 2:**
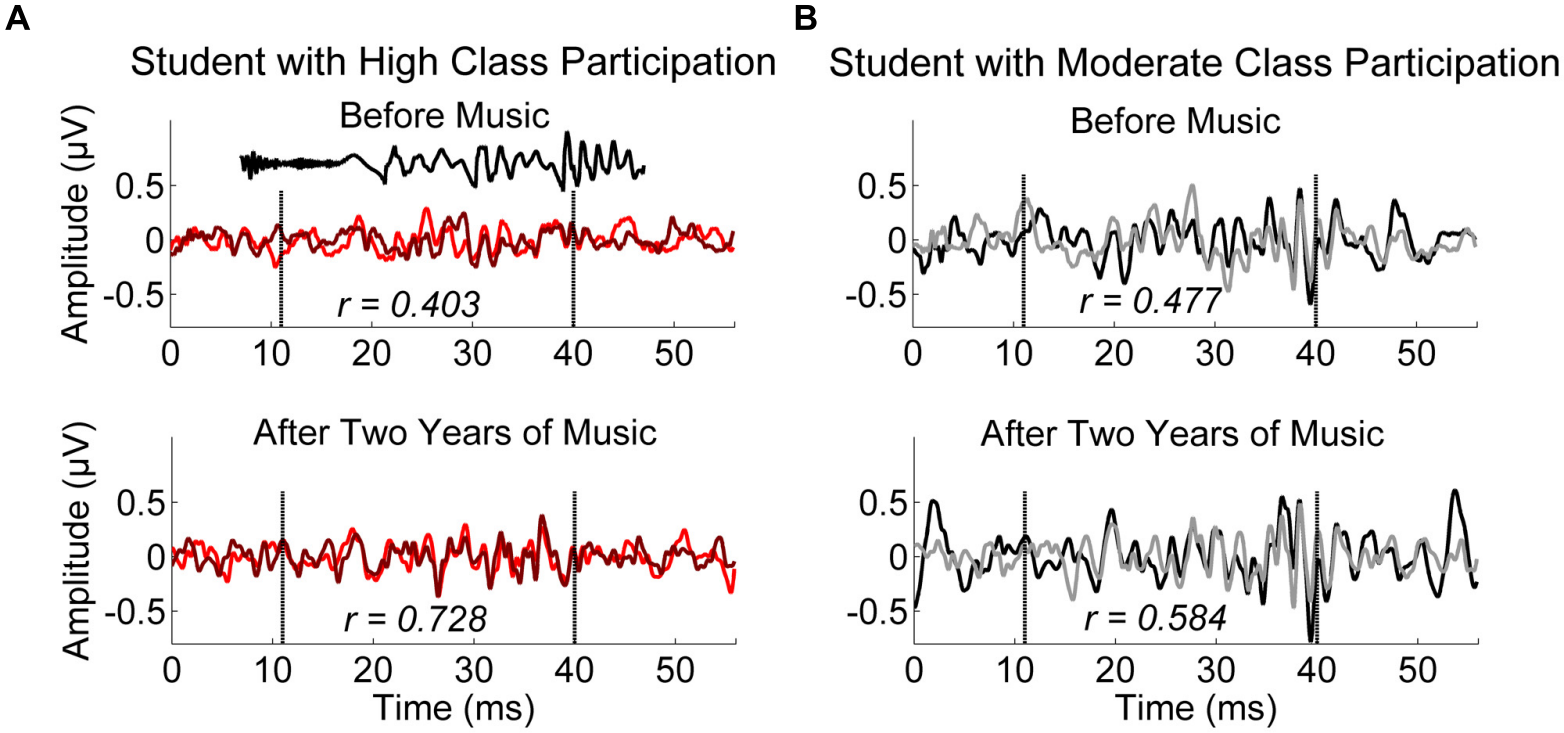
**Children who were most engaged during instrument classes had more consistent neural responses to speech after 2 years. (A)** (red/maroon), a representative subject who had “complete” participation as rated by multiple teachers. The [da] stimulus is plotted in black in top panel of A for reference, shifted in time to account for neural delay. **(B)** (black/gray), a representative subject who had “moderate” participation as rated by multiple teachers. Both students completed four instrumental classes over the course of 2 years. Before music training, (top) the participants do not differ greatly in the consistency of their neural response to speech. After 2 years of music training (bottom), however, the child who participated more in class has a more consistent response to speech than the child who participated less. The two traces in each panel represent the two replications of the response, collected as the recording procedure dictates. The time region of the analysis is marked with vertical hashed lines in each panel.

Neural measures were those previously shown to index SES (Skoe et al., 2013). Measures of response consistency, speech harmonics, and spontaneous neural activity were generated in MATLAB (Mathworks, Natick, MA, USA). Response consistency reflects the replicability of the response over the first half and second half of the recording on a scale from -1 to 1. Values were Fisher-transformed for analyses, but Pearson’s *r*-values are shown in **Figures 1** and **2**. Speech harmonics comprise the average amplitude of the frequency following response over the first-formant range of the stimulus (264–656 Hz). Spontaneous neural activity reflects the amplitude of brainstem activity in the absence of the stimulus, and likely reflects neural noise. Please see Banai et al. (2009), Hornickel and Kraus (2013), and Skoe et al. (2013) for additional details.

### STATISTICAL ANALYSES

The relationships among instrumental class attendance, class participation, and neural measures were evaluated at each of the two test dates using Spearman’s correlations due to the non-normal distributions of the music engagement variables. Spearman’s correlations are more conservative than Pearson’s correlations because they reduce the impact of outlying data points and are most appropriate for smaller sample sizes. Similar to Pearson’s correlations, Spearman’s rho is a direct measure of effect size, reflecting the proportion of variance in the dependent variable (neural measures) accounted for by the independent variable (music engagement variables). Correlation coefficients with magnitudes of 0.1 are considered small effect sizes, 0.3 medium, and 0.5 large (Cohen, 1992). Reading fluency scores were compared using a paired *t*-test.

## RESULTS

Children who had better attendance in instrumental music classes over 2 years had stronger neural encoding of speech harmonics and better response consistency, two measures previously linked to SES (see **Figure 1**; **Table 2A**). Additionally, there was a positive association between class participation and response consistency (see **Figure 2** for two representative participants and **Table 2A**). Interestingly, the least engaged participants in the study still had “moderate” class participation, suggesting that even small variations in student engagement can have implications for success with music training. No relationship was found between music engagement and spontaneous neural activity.

**Table 2:** **(A)** Children who are more engaged in music classes have stronger neural encoding of speech after music training for measures previously linked to SES (speech harmonics and response consistency). **(B)** Neural measures before beginning musical training do not predict subsequent engagement in music classes.

	Music engagement	
	Percent attendance	Class participation



**A**
Speech harmonics	**0.385 (0.052)**	0.242 (0.235)
Response consistency	**0.391 (0.048)**	**0.389 (0.049)**
Spontaneous neural activity	-0.201 (0.326)	0.013 (0.949)
**B**
Speech harmonics	-0.150 (0.464)	0.119 (0.562)
Response consistency	0.280 (0.166)	0.138 (0.502)
Spontaneous neural activity	-0.308 (0.126)	-0.212 (0.299)

*Values are Spearman’s *rho* (*p*-value). Significant relationships are bolded*.

Importantly, our results suggest that greater engagement in music classes predicts stronger speech encoding, not vice versa. Neural measures before music training did not predict subsequent attendance or class participation (*p*-values > 0.126, see **Table 2B**), which suggests that participants with strong speech encoding to begin with did not necessarily go on to be the most engaged students in the study. Additionally, the small variation in years of maternal education did not predict attendance or class participation (ρ = -0.302, *p* = 0.142; ρ = 0.126, *p* = 0.548).

Participants overall showed a subclinical yet statistically-significant decline in reading fluency over the 2 years [M1(SD1) = 109.27(12.76), M2(SD2) = 106.04(15.22), *t*_25_ = 2.456, *p* = 0.021]. However, change in reading fluency scores was strongly correlated with engagement in instrumental classes. Children who participated more in class were more likely to show an improvement in reading fluency, while those who participated less were more likely to show a decrease in reading fluency (ρ = 0.443, *p* = 0.023).

## DISCUSSION

Here we found that greater engagement in group music instruction by children from low SES backgrounds predicted stronger neural encoding of speech for measures negatively influenced by low SES. Children who attended class more regularly and had better classroom participation had stronger neural encoding of speech after 2 years of music training than did their less-engaged peers. These results were found *within* a group of children undergoing music training, and without an active control group. It is likely that greater engagement in other extracurricular activities could also yield stronger neural outcomes. Perhaps the novel experience of participating in an extracurricular activity, interacting with the researchers, etc., influenced our findings. However, previous studies that have employed active control groups indicate that music training but not art training strengthens auditory skills (Moreno et al., 2009, 2011). Moreover, child musicians differ from non-musician children on auditory working memory and attention but not visual analogs (Strait et al., 2014). Similar to our analyses within a group of children participating in music, Hyde et al. (2009) found that the quality of musical training is critical for neuroplasticity. Children participating in private keyboard lessons for 15 months showed structural growth in primary auditory cortex, correlated with their improvement in melody and rhythm perception tasks; however, a second group of children who participated in school music classes each week learning basic singing and drumming skills did not show the same neural growth (Hyde et al., 2009). Our results suggest that engagement is an important factor mediating the benefits seen from musical training. It’s important to note that we found these relationships between engagement and neural function within a group of students whose attendance was relatively high on average (88%), who were rated as having ‘moderate’ or better class participation, and whose families were highly motivated for them to participate. The limited variance in music engagement and probable influence of other factors such as personality on student engagement may have contributed to the modest statistical significance of our tests. Nevertheless, we do see moderately strong relationships between engagement and neural measures, suggesting that even if well-motivated to begin music training, students may not make gains unless they are actively engaged in the process. In the same vein, previous studies of musicians have revealed the important role of continued practice in maintaining the benefits of musicianship (Pantev et al., 1998; Ho et al., 2003; Norton et al., 2005; Forgeard et al., 2008b; see Strait and Kraus, 2014 for a review).

Greater attendance and class participation were not predicted by neural measures before musical training. This suggests that children with the strongest neural function before beginning musical practice did not go on to be those most engaged in music classes. Instead, it appears that greater engagement in music may have resulted in stronger neural encoding. Cohort research revealing neural differences between musicians and non-musicians is unable to definitively say whether those differences were pre-existing, although the oft observed relationship between length of practice and benefits of musicianship supports music experience as the cause (Pantev et al., 1998; Ho et al., 2003; Norton et al., 2005; Forgeard et al., 2008b; Seither-Preisler et al., 2014; see Strait and Kraus, 2014 for a review). A host of recent longitudinal studies report that musical training can selectively enhance auditory function in children without pre-existing differences. When children are randomly assigned to music or art training, only those in musical training show enhancements in auditory neurophysiology and attention (Moreno et al., 2009, 2011; Chobert et al., 2014). Additionally, children who elect to participate in music do not have inherent differences in neural structure or function from their peers (Norton et al., 2005; Hyde et al., 2009; Tierney et al., 2013), but show enhancements in auditory system structure and function after 1–2 years of musical training (Hyde et al., 2009; Tierney et al., 2013; Kraus et al., 2014b). We were not able to conduct paired pre-post comparisons due to alterations to our recording scheme as the study progressed. However, the lack of relationship between pre-test neural measures and subsequent classroom engagement in our study suggests our results are independent of pre-existing differences in neural function.

Although some studies report no differences in cognition for children who elect to participate in music versus those who do not (Schlaug et al., 2005; Hyde et al., 2009), personality and pre-existing skill may nevertheless influence a student’s engagement and persistence in music. For example, using duration of music lessons as the outcome variable, Corrigall et al. (2013) showed that cognitive skills (along with parental income) predicted how long students continued with music. They additionally showed that children exhibiting high Openness to experience (part of the Big Five personality traits) were more likely to continue with music lessons after taking into account cognition and parental income (Corrigall et al., 2013). In our group of students, personality factors may have influenced attendance and participation in music classes. Those who were highly engaged in music classes may have been highly engaged throughout their school day, had a more encouraging home environment, may have been more motivated individuals, etc. Indeed, Openness as a personality trait is linked to factors reflecting academic engagement and achievement in college students, such as enjoying thinking and analyzing, enjoying connecting with others in class, and enjoying working hard (Komarraju and Karau, 2005). One limitation of our study is the lack of measures to investigate which of these personality factors may predict engagement in music. Importantly though, duration of musical experience also predicts cognition and language skills (Schellenberg, 2006; Forgeard et al., 2008b), suggesting the presence of a reinforcing loop. Children more open to new experiences may elect to begin music lessons and continue to participate longer, which leads to larger cognitive benefits, predicting continued retention in music, etc.

In addition to enhancing cognitive skills, music training can lead to stronger reading, language, and academic skills in children (Schellenberg, 2004, 2006; Hetland and Winner, 2010; Corrigall and Trainor, 2011; Dege and Schwarzer, 2012; Francois et al., 2013; Seither-Preisler et al., 2014; Slater et al., 2014a; see Tierney and Kraus, 2013 for a review), including those with reading impairments (Overy, 2003; Bhide et al., 2013). As a group our participants showed a small decline in reading fluency scores, which likely reflects a trend for the achievement gap to widen for children from low SES backgrounds as they age (Sirin, 2005). However, the change in reading scores varied with music engagement; reading fluency improved in children with greater class participation. That this relationship was seen despite the small variance in class participation ratings speaks to the importance of active engagement in music for engendering benefits. Students who had the highest class participation rankings were more likely to show an increase in reading score after 2 years of participation in Harmony Project. On the other hand, participants who had ‘moderate’ class participation on average were likely to show a decrease in reading score. As the consistency of the auditory brainstem response to speech and the strength of representations of the speech harmonics have been repeatedly linked to reading ability (Banai et al., 2009; Hornickel et al., 2011, 2012a; Hornickel and Kraus, 2013; White-Schwoch and Kraus, 2013), and musical aptitude can predict reading ability (Ho et al., 2003; Forgeard et al., 2008a; Huss et al., 2011; Strait et al., 2011), it is possible that stronger speech encoding arising from greater participation in music classes contributed to improved reading scores.

Animal models of auditory deprivation and enrichment yield insight into the potential mechanisms involved in the neuroplasticity observed here. Similar to humans exposed to chronic noise, animals reared in noisy environments show detrimental effects on auditory processing, such as broader neural frequency selectivity (Zhou et al., 2011; Zhu et al., 2014), greater spontaneous neural activity (Seki and Eggermont, 2003), and weaker neural speech processing (Reed et al., 2014). Additionally, rats with a knock-down of a dyslexia-linked gene show more variable cortical responses to speech and weaker neural differentiation of speech (Centanni et al., 2014a). These deficits are thought to be due to imbalances in excitatory and inhibitory neurotransmitters, also shown to be negatively affected by noise exposure during development (Guo et al., 2012; Zhu et al., 2014). Auditory enrichment and behavioral training can reverse these effects, yielding better neural frequency selectivity, more consistent neural responses to speech, better neural differentiation of speech sounds, and can re-establish excitatory/inhibitory balances in rats with early auditory deprivation (Guo et al., 2012; Centanni et al., 2014b; Zhu et al., 2014). Although these exact mechanisms have not been investigated in humans, it is very likely that similar ones are at play. Like the auditory enrichment paradigms in animal studies, music training is a social, multi-sensory process that activates multiple neural networks during repeated exposure to the enriching “environment” [please see Patel (2011) for a review of the multi-faceted nature of music training].

We failed to find significant relationships between spontaneous neural activity and musical engagement. Previous studies of auditory training in humans, such as music training, computer-based training games, foreign language learning, and classroom acoustic modification, have shown improvements in the evoked response to speech, but none have reported changes in spontaneous neural activity (Besson et al., 2007; Song et al., 2008; Stevens et al., 2008; Russo et al., 2010; Hornickel et al., 2012b; Strait et al., 2013). Animals that are subjected to noise during development have increased spontaneous neural activity, likely due to decreased neural inhibition as a result of abnormal excitatory/inhibitory neurotransmitter ratios (Seki and Eggermont, 2003; Zhu et al., 2014). However, auditory enrichment can alleviate imbalances in excitatory and inhibitory neurotransmitters (Guo et al., 2012; Zhu et al., 2014) and possibly reduce noise-induced spontaneous neural activity. Spontaneous cortical activity thought to reflect a functional “resting-state network” can be altered with training in humans (Lewis et al., 2009; Taubert et al., 2011). The present findings show that the extent of music engagement tracks with changes in stimulus-evoked, but not spontaneous background activity; training-related influences on spontaneous activity should be explored further.

Our results support the importance of active experience and meaningful engagement with sound to engender neural changes. Even in a group of highly motivated students, small variations in music engagement (attendance and class participation) predicted the strength of speech encoding after music training. These measures of neural encoding are known to be weaker in children from low SES backgrounds. Although personality features likely played a large role in students’ levels of engagement in class, our results do support that greater levels of engagement are not prompted by pre-existing differences in neural encoding. In our group of participants from low SES backgrounds, those who were the most engaged in music classes had the strongest neural responses and were most likely to show improvements in reading ability. Community music programs such as Harmony Project and El Sistema have proven success in bolstering academic achievement in children from disadvantaged backgrounds. We have also shown that participation in Harmony Project reinforces literacy skills and enhances the neural encoding of speech cues important for reading and the perception of speech in noisy backgrounds (Kraus et al., 2014a,b; Slater et al., 2014a; Kraus and Strait, in press). Taken together, we suggest that motivated engagement in community music programs may counteract some of the auditory impoverishment that children from low SES backgrounds commonly experience.

## AUTHOR CONTRIBUTIONS

Nina Kraus and Dana L. Strait designed the study; Dana L. Strait, Jessica Slater, and Elaine Thompson collected the data; Jane Hornickel analyzed the data; Jane Hornickel and Nina Kraus prepared the manuscript; all authors contributed to the final version of the manuscript.

## Conflict of Interest Statement

The authors declare that the research was conducted in the absence of any commercial or financial relationships that could be construed as a potential conflict of interest.
